# A Robust Hierarchical MXene/Ni/Aluminosilicate Glass Composite for High‐Performance Microwave Absorption

**DOI:** 10.1002/advs.202104163

**Published:** 2021-12-13

**Authors:** Wei Luo, Mengya Wang, Kangjing Wang, Peng Yan, Jilong Huang, Jie Gao, Tao Zhao, Qi Ding, Pengpeng Qiu, Haifeng Wang, Ping Lu, Yuchi Fan, Wan Jiang

**Affiliations:** ^1^ State Key Laboratory for Modification of Chemical Fibers and Polymer Materials College of Materials Science and Engineering Donghua University Shanghai 201620 China; ^2^ Institute of Functional Materials Donghua University Shanghai 201620 China; ^3^ State Key Laboratory of High Performance Ceramics and Superfine Microstructures Shanghai Institute of Ceramics Chinese Academy of Sciences Shanghai 200050 China

**Keywords:** hierarchical glass composite, mechanical properties, microwave absorption, MXene, sintering

## Abstract

The 2D titanium carbide MXene with both extraordinary electromagnetic attenuation and elastic properties has shown great potential as the building block for constructing mechanically robust microwave absorbing composites (MACs). However, the weak thermal stability has inhibited the successful incorporation of MXene into the inorganic MACs matrix so far. Herein, an ultralow temperature sintering strategy to fabricate a hierarchical aluminosilicate glass composite is demonstrated by using EMT zeolite as starting powder, which can not only endow the composites with high sinterability, but also facilitate the alignment of MXene in the glass matrix. Accordingly, the highly oriented MXene and mesoporous structure can effectively reduce the conduction loss in the out‐of‐plane direction while maintaining its large polarization loss. Meanwhile, the in situ formed Ni nanoparticles via ion exchange serve as a synergistic modulator to further improve the attenuation capability and impedance matching of composite, resulting in a low reflection loss of −59.5 dB in X band and general values below −20 dB with a low fitting thickness from 4 to 18 GHz. More attractively, such a delicate structure also gives the composite a remarkable fracture strength and contact‐damage‐resistance, which qualifies the mesoporous glass composite as a structural MACs with a superior comprehensive performance.

## Graphical abstract

A hierarchical aluminosilicate glass composite incorporated with aligned MXene nanosheets and isolated Ni nanoparticles is constructed via in situ reaction and low‐temperature sintering of EMT zeolite. The unique structure resolves the trade‐off between attenuation and impedance matching to give high microwave absorbing performance, while providing remarkable mechanical properties in terms of fracture strength, hardness, and contact damage resistance.

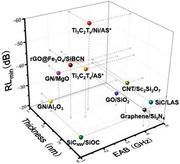

## 1 Introduction

With the rapid development of modern telecommunication technologies, especially the advent of the fifth generation wireless system, electromagnetic interference from the microwave frequencies has become a ubiquitous source of pollution, which not only causes malfunction to the operation of electronic devices, but also directly affects the public health.^[^ ^1^ ^]^ Therefore, the exploration of high‐performance microwave absorbing (MA) materials is of great importance in controlling the environmental hazard from the microwave radiation through consuming the majority of electromagnetic energy inside the material.^[^ ^2^, ^3^ ^]^ Considering the ideal design criteria for the modern MA materials including lightweight, low thickness, and broad absorbing, the 2D materials with a large surface area and a high attenuation capability are very attractive candidate absorbents.^[^ ^4^, ^5^ ^]^ In particular, the transition metal carbide MXene represented by Ti_3_C_2_T*_x_* with a high conductivity and polarization loss has been proved to possess enormous efficiency in microwave shielding.^[^ ^1^, ^6^ ^]^ However, the extremely large permittivity induced poor impedance matching renders MXene material overwhelmingly reflective feature to the incident microwave, even in the forms of foam or aerogel.^[^ ^7^, ^8^ ^]^ To improve the impedance matching of MXene, constructing MA composites (MACs) with a matrix of low dielectric loss is generally accepted. It is found that the very low fraction of MXene can lead to a significant improvement of MA performance due to its high attenuation capability.^[^ ^9^, ^10^, ^11^ ^]^ However, the optimization of the MA properties by varying the content of MXene is still difficult due to the suddenly increased conduction loss after percolation. For instance, the MXene/Ni chain hybrid mixed with paraffin has a minimum reflection loss (RL_min_) of −49.9 dB at 11.9 GHz when the MXene content is 10 wt% with respective to Ni, while it could change into a highly reflective shielding material (≈80% reflection) as the MXene content rises to 20 wt%.^[^ ^12^ ^]^ Therefore, searching for a more effective modulating strategy is very urgent in the development of MXene based MACs. Moreover, the previously reported MXene based MACs can hardly provide satisfactory mechanical strength to withstand the applied loading. To this end, bulk composites with a fully inorganic matrix are highly desirable for the practical applications. Particularly, the silicate glass with a low density, a high microwave transparency, and an excellent corrosion resistance as well as a strong mechanical strength at an elevated temperature distinguishes itself as a perfect matrix for mechanically robust MACs. Unfortunately, the carbide MXene is not sufficiently stable to survive from the high‐temperature treatment applied for the preparation of silicate glass (normally > 1300 ℃).^[^ ^13^ ^]^ Our recent research has demonstrated that the densification temperature can be lowered down to 1020 ℃ by using a mesoporous powder (e.g., SBA‐15, MCM‐41) as the sintering sources.^[^ ^14^, ^15^ ^]^ However, it is still much higher than the temperature (<800 ℃) that can prevent the MXene from degradation in the composites.^[^ ^16^ ^]^

Herein, a well‐designed Ti_3_C_2_T*_x_* @EMT‐Ni zeolite composite with a core–shell structure was exploited as the raw powder to fabricate a mesoporous MXene/Ni/aluminosilicate (AS) glass composite by using a popular spark plasma sintering (SPS) technology. EMT is a zeolite material with a Si/Al ratio of 1.14, which can facilely grow on the MXene nanosheets and further be ion‐exchanged with Ni ions.^[^ ^17^ ^]^ The derived composites show a very high sinterability, which can form a AS glass at an ultralow temperature (700 ℃), thus preserving the Ti_3_C_2_T*_x_* nanosheet in the composite from thermal damage. The viscous flow induced by the highly oriented Ti_3_C_2_T*_x_* nanosheet in matrix effectively alleviate the high conduction loss in the out‐of‐plane direction for achieving a better impedance matching. Note the out‐of‐plane direction here refers to the direction along the thickness of bulk sample. Meanwhile, the porous channel structure for cations can be exploited for uniformly introducing the third phase of Ni NPs for further tuning the electromagnetic properties of composite. The MA performance and mechanical properties of the composite were investigated and corresponding mechanisms were discussed.

## 2 Results and Discussion

### 2.1 Microstructure of the Mesoporous Glass Composite

**Figure 1:**
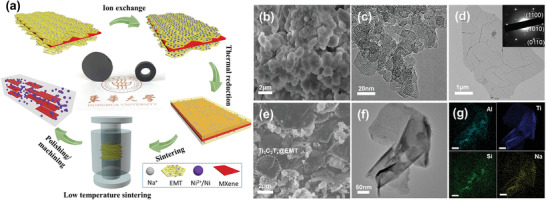
a) Schematic illustration for the fabrication of Ti_3_C_2_T*_x_*/Ni/AS composite. b) SEM and c) TEM images of EMT powder, inset in (c) is the high resolution TEM image of EMT NPs, scale bar: 2 nm. d) TEM image of Ti_3_C_2_T*_x_* nanosheet, inset is the corresponding SAED pattern. e) SEM, f) TEM, and g) corresponding EDS mapping images of Ti_3_C_2_T*_x_* @EMT hybrid powder, scale bar in (g) is 50 nm.

The mesoporous Ti_3_C_2_T*_x_*/Ni/AS glass composite was derived from the ultralow temperature sintering of a hybrid Ni ion‐exchanged EMT zeolite/MXene composites (**Figure** **1**a). The EMT powder was prepared through a template‐free method, which shows a sphere‐like morphology with clear hexagonally compacted channels under TEM observation (Figure 1b,c). The exfoliated MXene with a lateral size of several micrometers is thin enough to form wrinkles on the surface (Figure 1d), while still maintaining high in‐plane crystallinity as indicated by selected area electron diffraction (SAED). Owing to the large flat surface of Ti_3_C_2_T*_x_* nanosheets, the EMT zeolite NPs can be easily grown on the surface of MXene (Ti_3_C_2_T*_x_* @EMT) after adding the MXene as one precursor (Figure 1e). The TEM image clearly shows that the MXene nanosheets become thickening (Figure 1f). The EDS elemental mapping images (Figure 1g) display the uniform distribution of Al, Si, Ti, and Na elements on the entire Ti_3_C_2_T*_x_* @EMT composite nanosheets, further confirming the successful coating of EMT zeolite on Ti_3_C_2_T*_x_* nanosheet.

**Figure 2:**
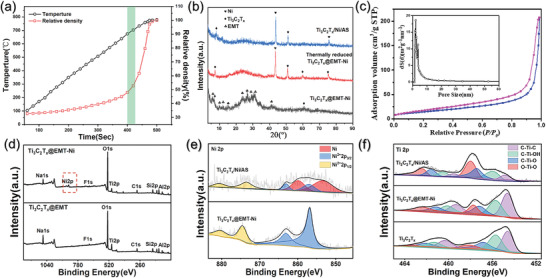
a) The correlation between relative density and temperature with increasing sintering time for AS glass. b) XRD patterns of Ti_3_C_2_T*_x_* @EMT‐Ni powder, thermally reduced Ti_3_C_2_T*_x_* @EMT‐Ni powder, and Ti_3_C_2_T*_x_*/Ni/AS composite. c) Nitrogen sorption isothermals of the Ti_3_AlC_2_/Ni/AS composites, insert shows the pore size distribution derived from the adsorption data. d) XPS survey spectra of Ti_3_C_2_T*_x_* @EMT and Ti_3_C_2_T*_x_* @EMT‐Ni powder. e) Ni 2p peak of XPS for Ti_3_C_2_T*_x_* @EMT‐Ni and thermally reduced Ti_3_C_2_T*_x_* @EMT‐Ni powders. f) Ti 2p peak of XPS for Ti_3_C_2_T*_x_*, thermally reduced Ti_3_C_2_T*_x_* @EMT‐Ni powder and Ti_3_C_2_T*_x_*/Ni/AS composite.

The ion exchangeable feature of EMT enables the homogeneous incorporation of metallic NPs such as Ni in the AS matrix, which is critical to the optimization of electromagnetic properties. After exchange of Ni^2+^ cation, the hybrid powder (denoted as Ti_3_C_2_T*_x_* @EMT‐Ni) was subjected to a thermal reduction for generating metallic Ni NPs, during which the zeolite structure was collapsed and transformed into amorphous phase completely (Figure S1a, Supporting Information) due to the vulnerable thermal stability of EMT. Surprisingly, it is found that the EMT derived amorphous powder still exhibited very high sinterability despite the decreased surface area, given the fact that the fully dense AS glass can be achieved by SPS at temperature as low as 780 ℃ (**Figure** **2**a). It is deduced that the high sinterability is attributed to the ultrafine gain size (≈20 nm) of the AS powder formed after heat treatment (Figure S2, Supporting Information). Meanwhile, the thermal stability of Ti_3_C_2_T*_x_* nanosheet under the circumstance of oxide glass matrix must be considered. As indicated by the XRD patterns (Figure 2b), the presence of the typical peak of (002) plane in Ti_3_C_2_T*_x_* can be observed after thermal reduction and sintering until 700 ℃, while completely disappearing when the sintering temperature is higher (Figure S1b, Supporting Information). Thus, the densification temperature for composite was determined to be 700 ℃, which leads to a relative density similar to 50% for the AS glass. It has to be noted that the glass composites obtained at this condition possess a mesoporous structure (Figure 2c), which is essential in terms of lowering the permittivity of matrix for proper impedance matching.^[^ ^18^ ^]^ The presence of mesoporous structure was evaluated by the nitrogen adsorption−desorption isotherms, which show a typical type IV curve with a H_2_‐type hysteresis loop, in reflection of the existence of mesopores. The BET surface area and pore size were calculated to be 58.4 m^2^ g^−1^ and 3.7 nm, respectively. It is deduced that the pores were formed due to the grain growth during initial and intermediate stage of sintering. In addition, after heat treatment at 550 °C in Ar/H_2_ forming gas, the peaks assigned to metallic Ni (PDF:45‐1027) can be identified for heat treated Ti_3_C_2_T*_x_* @EMT‐Ni powder and corresponding bulk Ti_3_C_2_T*_x_*/Ni/AS composite, indicating the effective reduction of exchanged Ni^2+^. For comparison, a Ni‐free Ti_3_C_2_T*_x_*/AS composite was also prepared by densification of Ti_3_C_2_T*_x_* @EMT powder without ion exchange (Figure S1c, Supporting Information).

The composition evolution during processing can be monitored by X‐ray photoelectron spectroscopy (XPS) spectra. The survey spectra show that after ion exchange Ni was introduced to the EMT, but Na was not completely removed from the skeleton (Figure 2d). In the high resolution XPS peak of Ni 2p (Figure 2e), the fitted components assigned to zero‐valent state of Ni (852.6 and 858.7 eV) becomes dominant after thermal reduction, which confirms the generation of metallic Ni NPs.^[^ ^19^ ^]^ Accordingly, around 57% of Ni^2+^ has been reduced to Ni^0^, whose amount in composite can be manipulated by the concentration of Ni^2+^ in solution. In addition, the quality of Ti_3_C_2_T*_x_* nanosheet can be revealed by the Ti 2p signals (Figure 2f), which could be fitted into four components corresponding to O—Ti—O (463.98 and 458.1 eV), C—Ti—O (462.6 and 457.5 eV), C—Ti—OH (461.3 and 455.5 eV), and the retained C—Ti—C bond (460.0 and 454.5 eV) species, respectively.^[^ ^20^ ^]^ The detected O—Ti—O peak verifies the oxidation on Ti_3_C_2_T*_x_* right after the in situ coating of EMT_,_ which continued even during thermal reduction and sintering despite the highly reductive environment. However, most of C—Ti–T*_x_* components were preserved in the composite, indicating that in‐plane structure of Ti_3_C_2_T*_x_* nanosheet could withstand the high temperature during SPS in large extent.

**Figure 3:**
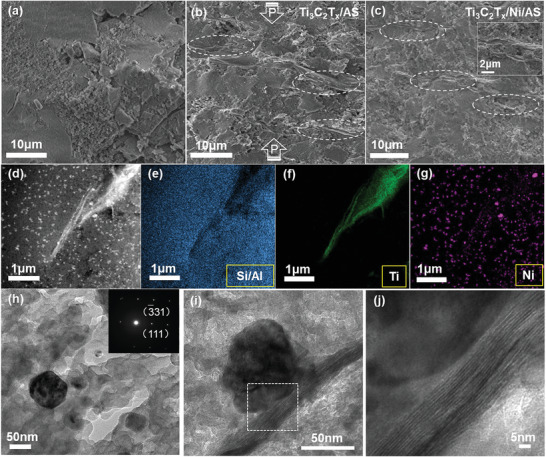
a–c) SEM images of fracture surface for AS, Ti_3_C_2_T*_x_*/AS composite, and Ti_3_C_2_T*_x_*/Ni/AS composite, respectively; the arrows in (b) indicate the direction of hot‐pressing. d) HAADF–STEM image and e–g) corresponding EDS elemental mapping of Ti_3_C_2_T*_x_*/Ni/AS composite, respectively. h) TEM image showing a Ni particle in glass matrix, inset is the corresponding SAED pattern. i) TEM image showing the in situ formed Ni nanoparticle on Ti_3_C_2_T*_x_* nanosheet in Ti_3_C_2_T*_x_*/Ni/AS composite. j) HRTEM image for the area marked by dash square in (i).

The effect of densification can be directly confirmed from the fracture surface of sintered samples. In the pure AS glass, the fracture surface exhibits a very rough feature (**Figure** **3**a) compared to the fully densified glass (Figure S3, Supporting Information), which reflects the large amount of residual pores in the bulk glass sample (*ρ*_r_ = 50%). With the addition of MXene, the fracture surface of Ti_3_C_2_T*_x_*/AS composite seems rougher compared to the AS glass due to the presence of second phase, though the relatively density is almost same (Figure 3b and Table S1, Supporting Information). The fractured Ti_3_C_2_T*_x_* nanosheet can be distinguished clearly from the glass matrix due to the characteristic laminate structure. In the presence of Ni, the microstructure of Ti_3_C_2_T*_x_*/Ni/AS composite shows no noticeable difference compared with the Ni‐free composite. Thanks to the pronounced laminate structure of MXene, it is found that there is very obvious alignment of Ti_3_C_2_T*_x_* nanosheets in the AS glass matrix perpendicular to the pressing direction of sintering. Noting that such highly oriented alignment of nanosheets is not commonly observed in other 2D materials containing composite prepared by hot‐pressed sintering,^[^ ^13^ ^]^ we deduce that the low softening point and corresponding transient viscous flow of AS glass played a key role during the sintering process, which facilitated the alignment of 2D Ti_3_C_2_T*_x_* nanosheets in matrix under uniaxial pressure.

The unique microstructure in Ti_3_C_2_T*_x_*/Ni/AS composite was further revealed by STEM and TEM analysis. The HAADF–STEM image (Figure 3d) shows two types of inclusions in the matrix with brighter contrast. The one with a layered paper‐like morphology is Ti_3_C_2_T*_x_* nanosheet, which can be confirmed by the EDS mapping from the concentrated Ti element (Figure 3f). The other type of inclusion displays as bright dots in the AS glass matrix under low magnification, which can be identified as Ni NPs according to both elemental mapping and SAED (Figure 3g,h). The isolated Ni NPs should be generated due to the reduction of Ni^+^ ions trapped inside the wall of channels in EMT. The Ni content in the matrix can be manipulated by the extent of ion exchange very well (Figure S4, Supporting Information), and the NPs with size lower than 50 nm distribute homogeneously in the glass matrix without any noticeable agglomeration even at high concentration (4.7 vol%), highlighting the great advantage of the strategy based on ion‐exchange and in situ reduction. Since the Ni NPs are randomly distributed in the matrix, they have the chance to contact with MXene nanosheets and form Ni/Ti_3_C_2_T*_x_* heterointerfaces (Figure 3i,j), which may have great influence on the microwave attenuation capability of composite.

### 2.2 Electromagnetic and MA Properties of the Mesoporous Glass Composite

**Figure 4:**
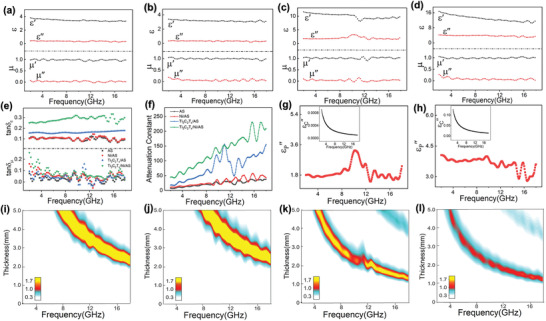
a–d) Complex permittivity of AS glass, Ni/AS, Ti_3_C_2_T*_x_*/AS, and Ti_3_C_2_T*_x_*/Ni/AS composites, respectively. e) Frequency dependence of tan*δ_*ε*_* and tan*δ_µ_* for various samples. f) Frequency dependence of attenuation constant for various samples. g,h) Frequency dependence of $\varepsilon_{c}^{{}^{\prime \prime }}$ and $\varepsilon_{p}^{{}^{\prime \prime }}$for Ti_3_C_2_T*_x_*/AS and Ti_3_C_2_T*_x_*/Ni/AS composites, respectively. i–l) Contour maps of the *Z*_in_ for AS glass, Ni/AS, Ti_3_C_2_T*_x_*/AS, Ti_3_C_2_T*_x_*/Ni/AS composites, respectively.

The Ti_3_C_2_T*_x_* type MXene possesses high attenuation capability due to its high dielectric loss derived from both large real and imaginary permittivity.^[^ ^21^ ^]^ Therefore, the matrix material in the MXene incorporated composite should have low complex permittivity to optimize the impedance matching which can be described as follows

(1) $$Z_{\mathrm{in}}=\sqrt{\frac{\mu_{r}}{\varepsilon_{r}}}\;\tan h\left(j\frac{2\pi fd}{c}\sqrt{\mu_{r}\varepsilon_{r}}\right)$$

where *ε*_r_ and *μ*_r_ are the complex relative permittivity and permeability, respectively, *f* is the microwave frequency, *d* is the thickness of the absorbed layer, and c is the velocity of the electromagnetic wave in vacuum. Owing to the high porosity and low intrinsic dielectric constant, the complex permittivity of pure AS glass is very low for both real (*ε*′) and imaginary (*ε*″) parts, which gives rise to the dielectric loss around 0.1 throughout the investigated frequency from 2 to 18 GHz (**Figure** **4**a–c). Therefore, the AS glass can provide adequate space for tuning dielectric properties of MXene included composite. With addition of 10 wt% Ti_3_C_2_T*_x_* nanosheets (≈8.84 vol%), both *ε*′ and *ε*″ of Ti_3_C_2_T*_x_*/AS composite shows prominent increase. As a highly conductive 2D material, the Ti_3_C_2_T*_x_* nanosheet is highly efficient in enhancing the electrical conductivity of composites,^[^ ^22^ ^]^ which could result in very large *ε*″ according to the following relation

(2) $$\varepsilon^{\prime \prime }=\varepsilon_{p}^{{}^{\prime \prime }}\;+\;\varepsilon_{c}^{{}^{\prime \prime }}=\frac{\varepsilon_{s}-\varepsilon_{\infty }}{1+\omega^{2}\tau^{2}}\;\omega \tau +\frac{\sigma }{\omega \varepsilon_{0}}$$

where $\varepsilon_{p}^{{}^{\prime \prime }}$ and $\varepsilon_{c}^{{}^{\prime \prime }}$ represent the polarization loss and conduction loss, respectively; *ω* is the angular frequency, *τ* is the relaxation time, *σ* is the leakage conductivity, *ε*_s_ is the static permittivity, *ε*_∞_ is the relative dielectric permittivity at the high‐frequency limit. However, too large *ε*′′ also means severe impedance mismatching, which usually leads to highly reflective feature for the composite. As an example, the electrical conductivity as high as 1081 S m^–1^ can be achieved in polystyrene composite when the MXene content is 1.9 vol%, which gives rise to an excellent microwave shielding material with reflection effectiveness higher than 80%.^[^ ^20^ ^]^ In contrast, the out‐of‐plane electrical conductivity of Ti_3_C_2_T*_x_*/AS composite at low frequency limit is merely 7.6 × 10^–4^ S m^–1^ (Figure S5, Supporting Information), which is orders lower compared to the reported polystyrene composite. It is worth noting that the electrical conductivity of heating and sintering treated Ti_3_C_2_T*_x_* nanosheet has an electrical conductivity even higher than that of the pristine one due to the better crystallization (Figure S1d, Supporting Information). Accordingly, the dielectric loss can be considered as completely polarization induced in the Ti_3_C_2_T*_x_*/AS composite (Figure 4g), which is distinguished from any reported MXene based composites before. Moreover, the frequency dependent out‐of‐plane conductivity shows that the Ti_3_C_2_T*_x_*/AS composite is still not percolated in this direction (Figure S5, Supporting Information), while the in‐plane conductivity can be readily measured to be 0.58 S m^–1^, revealing the great anisotropy in the composite. Indeed, the low electrical conductivity can be partially ascribed to the mesoporous structure in AS matrix, which can separate the nanosheets more effectively than the fully dense matrix. However, it should be noted that the highly anisotropic structure of Ti_3_C_2_T*_x_* nanosheet also accounts for the low electrical conductivity, because the alignment of nanosheets is against the formation of conducing network in out‐of‐plane direction. Benefiting from this mesoporous and ordered structure, the Ti_3_C_2_T*_x_*/AS composite shows much improved impedance matching especially in 9–11 GHz compared with pure AS glass, despite the highly increased attenuation constant (Figure 4f) that can be calculated based on the equation

(3) $$\begin{array}{ccc} \alpha & = & \frac{\sqrt{2}\pi f}{c}\\ & & \times \sqrt{\left(\mu^{\prime \prime }\varepsilon^{\prime \prime }-\mu^{\prime }\varepsilon^{\prime }\right)+\sqrt{{\left(\mu^{\prime \prime }\varepsilon^{\prime \prime }-\mu^{\prime }\varepsilon^{\prime }\right)}^{2}+{\left(\mu^{\prime }\varepsilon^{\prime \prime }+\mu^{\prime \prime }\varepsilon^{\prime }\right)}^{2}}} \end{array}$$

However, it is difficult to improve both attenuation capacity and impedance matching by solely increasing the content of MXene, since the latter could become worse immediately after the percolation is achieved in the out‐of‐plane direction (Figure S6, Supporting Information). Therefore, instead of adding more MXene, 0D Ni NPs were introduced as a smart modulator among aligned Ti_3_C_2_T*_x_* nanosheets in the AS glass matrix. On one hand, the incorporation of the Ni NPs further enhanced the *ε*′ and *ε*′′, leading to increased tan*δ_*ε*_* to 0.2–0.3 which is favorable for increasing attenuation capability. On the other hand, the enhanced permittivity and permeability properly adjusted the impedance matching of composite as well. The *Z*_in_ value around unit can be found throughout the frequency range at proper thickness, which is critical to achieve high MA performance. More intriguingly, it is found that while incorporating Ni alone cannot induce any change for the permittivity in Ni/AS composite, the combination of Ti_3_C_2_T*_x_* nanosheets and Ni NPs together can largely increase *ε*′ and *ε*′′ with respect to the Ti_3_C_2_T*_x_*/AS composite, which mainly accounts for the highly improved attenuation constant. Note that the contribution from magnetic loss is relatively small for Ti_3_C_2_T*_x_*/Ni/AS composite owing to the Snoek limit of Ni.^[^ ^23^ ^]^

To elucidate this synergistic phenomenon, we further analyzed the imaginary permittivity from conduction loss and polarization loss in the Ti_3_C_2_T*_x_*/Ni/AS composite. The frequency dependent electrical conductivity indicates the composite is percolated via the in situ created Ni NPs, given the constant value in the entire low frequency range. However, the out‐of‐plane electrical conductivity is still very low (1.14 × 10^–2^ S m^–1^ for the composite with 3.7 vol% of Ni), which gives rise to small $\varepsilon_{c}^{{}^{\prime \prime }}$ (Figure 4h) with regard to *ε*′′. It is deduced that this limited increase of electrical conductivity mainly comes from the electron hopping effect mediated by isolated Ni NPs.^[^ ^24^ ^]^ In contrast, the in‐plane electrical conductivity has obvious change with the addition of Ni (Table S1, Supporting Information), because of the alignment of MXene nanosheets along this direction. As a result, the majority of enhanced dielectric loss can still be ascribed to the increase of $\varepsilon_{p}^{{}^{\prime \prime }}$. However, the Ni/AS composite with identical content of Ni NPs shows very similar permittivity and attenuation constant in comparison to the AS glass (Figure 4a,b,f), which excludes the Ni/AS interfacial polarization as the main origin of increased polarization loss. Therefore, it can be concluded that the polarization at Ni/Ti_3_C_2_T*_x_* heterointerface plays the most important role in the enhanced attenuation capability. Although the interfacial polarization effect between Ni and Ti_3_C_2_T*_x_* has been reported before,^[^ ^12^ ^]^ the quantitative result of this synergy has never been proved by experiment before due to the coupled factors. Herein by forming the highly anisotropic structure of MXene and isolated Ni particles in matrix, we unambiguously verified the synergistic effect at Ni/Ti_3_C_2_T*_x_* heterointerface for the first time.

**Figure 5:**
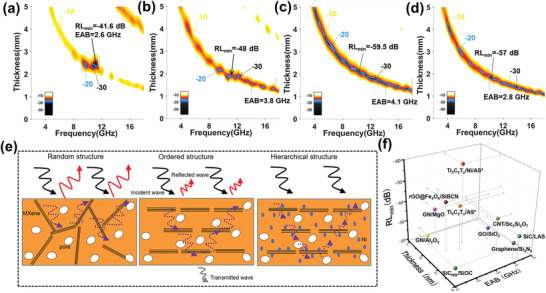
a–d) Contour maps of RL values with respect to frequency and thickness for Ti_3_C_2_T*_x_*/AS composite and Ti_3_C_2_T*_x_*/Ni/AS composite with Ni content of 1.8, 3.1, and 4.7 vol%, respectively. e) Different MA mechanisms for composites with random or ordered structure containing 2D absorber and mesoporous structure containing 2D/0D absorber. f) MA performance of typical inorganic matrix MACs compared with the values in this work (marked by stars), details can be seen in Table S2 in the Supporting Information.

With highly improved impedance matching and attenuation capability, the mesoporous Ti_3_C_2_T*_x_*/Ni/AS composite exhibits superior MA performance among the investigated samples. While the pure AS glass and Ni/AS composites both have negligible MA performance (Figure S7, Supporting Information), the Ti_3_C_2_T*_x_*/AS composite has the RL_min_ values of −41.65 dB at 10.5 GHz under the thickness of 2.35 mm (**Figure** **5**a). Besides, the effective absorbing bandwidth (EAB, the frequency range where RL values are lower than −10 dB at certain thickness) is calculated as 2.6 GHz from 8 to 12 GHz, reflecting the application potential in X band for this material. However, the RL values in other bands can hardly reach −10 dB in general, mainly due to the relatively poor impedance matching (Figure 4k). By tuning the content of Ni NPs, the strongest absorption reaches −59.5 dB at 10.8 GHz in Ti_3_C_2_T*_x_*/Ni/AS composite with 3.1 vol% Ni (Figure 5b–d). Meanwhile, the EAB of 3 GHz in X band and as wide as 4.1 GHz in Ku band can be achieved. It is found that although the isolated Ni NPs cannot further strengthen the attenuation capacity of composite, it affects the impedance matching greatly (Figure S8, Supporting Information) due to the magnetic nature of Ni. Very interestingly, it is found that the Ti_3_C_2_T*_x_*/Ni/AS composite with 3.1 vol% Ni shows the largest saturation magnetization and coercivity (Figure S9, Supporting Information), which can be ascribed to the smallest grain size among the three composites (Figure S4, Supporting Information). Accordingly, the RL value lower than −20 dB can always be satisfied under certain fitting thickness beneath 4.5 mm, which greatly broaden the applicable range as an effective microwave absorber. The high MA performance can be ascribed to two important factors, as illustrated in Figure 5. One is that the alignment of MXene nanosheets in matrix greatly improved the impedance matching while preserving the polarization loss of MXene. The other one is that the mesoporous structure with in situ formed Ni nanoparticles further optimized the impendences matching while enhancing the attenuation capacity. Considering the apparent density of 1.51 g cm^–3^ (Table S1, Supporting Information) for Ti_3_C_2_T*_x_*/Ni/AS composite, the specific MA performance is one of the best among reported inorganic MACs (Figure 5f and Table S2, Supporting Information).^[^ ^25^, ^26^, ^27^, ^28^, ^29^, ^30^, ^31^, ^32^ ^]^

### 2.3 Mechanical Properties of the Mesoporous Glass Composite

**Figure 6:**
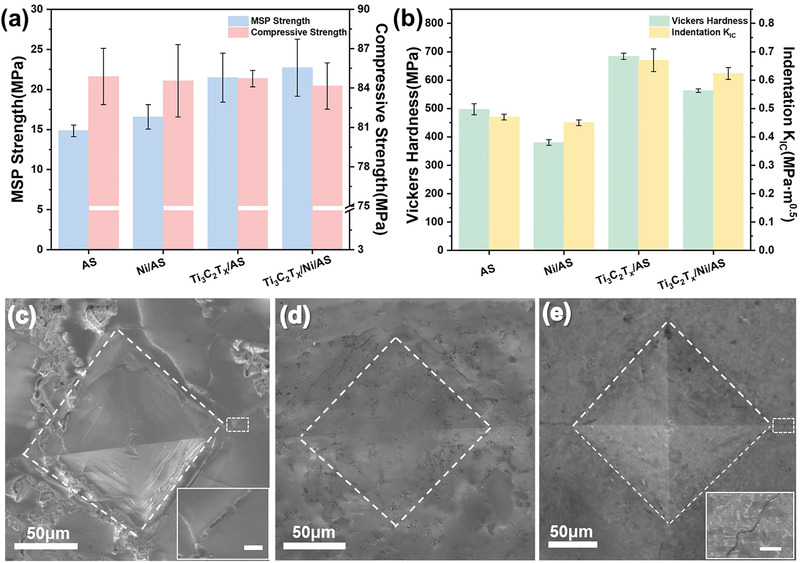
a) MSP and compressive strength for various samples. b) Young's Modulus and Vickers hardness for various samples. c–e) SEM images of indent under 1 kgf for AS glass and Ti_3_C_2_T*_x_*/Ni/AS composite, and the indent under 3 kgf for Ti_3_C_2_T*_x_*/Ni/AS composite, respectively; the insets in (c,e) show the magnified images of cracks with scale bar of 5 µm.

Compared to the powder type MACs, the unparalleled advantage of bulk MACs lies in their superior mechanical properties against deformation. As shown in **Figure** **6**a, the zeolite derived AS glass has a compressive strength higher than 80 MPa, which is robust enough for most applications. The compressive strength is much better than many lightweight MACs ^[^ ^33^, ^34^ ^]^ and even some porous ceramics,^[^ ^35^ ^]^ considering the high porosity in the AS glass. The addition of Ti_3_C_2_T*_x_* nanosheets and Ni NPs seems to have no obvious influence on the compressive strength, indicating the load bearing capacity under compression is mainly determined by the porosity rather than microstructures. However, the introduced MXene shows obvious enhanced flexural strength by using the modified small punch (MSP) test, which is a convenient bending test giving values similar to four‐point bending for small specimens.^[^ ^36^ ^]^ The MSP strength improves from 14 to 22 MPa when 10 wt% of MXene is added, which can be ascribed to the effective load transfer at Ti_3_C_2_T*_x_*/AS interface and preferred orientation of 2D Ti_3_C_2_T*_x_* in the matrix. For the Ti_3_C_2_T*_x_*/Ni/AS composite, the measured MSP strength is slightly higher compared with the Ti_3_C_2_T*_x_*/AS composite, suggesting the influence from 0D Ni particles as well. Given that the Ni/AS composite also shows higher MSP strength with respect to the AS glass, it is very likely that the dispersion strengthening mechanism accounts for the enhanced fracture strength.

Moreover, it is found the composites also possess impressive hardness and contact‐damage‐resistance compared to the porous AS glass, which is very attractive when applied as the outmost layer of devices. The introduction of MXene can greatly improve the Vickers hardness from 500 to 700 MPa for the porous AS glass (Figure 6b), which could be attributed to the high elastic modulus of Ti_3_C_2_T*_x_*.^[^ ^37^ ^]^ Although the hardness of composite decreases slightly with the presence of Ni, the value remains overwhelmingly higher compared to the traditional polymers such as polystyrene and poly(methyl methacrylate),^[^ ^38^ ^]^ highlighting the advantage of inorganic MACs. Moreover, while the AS glass shows classical radial cracks around the corner of indent even under relatively low loading force (1 kgf) (Figure 6c), no cracks can be found in the mesoporous Ti_3_C_2_T*_x_*/Ni/AS composite under the same loading force (Figure 6d), revealing the remarkable resistance upon contact damage. Similarly, the indentation toughness measured using higher loading is also much better than that of pure AS glass, which can be understood from the toughening mechanism of 2D materials. Specifically, the 2D MXene can effectively deflect the crack propagation while dissipating more fracture energy to increase the toughness of glass matrix. It can be seen in the AS glass that the indentation induced crack propagate straightly without any deflection, while in the Ti_3_C_2_T*_x_*/Ni/AS composite the crack underwent obvious deflection to form a zigzag pattern on the surface (Figure 6e). Since the composite contains no large grain in the matrix, this crack deflection can only be ascribed to the presence of MXene.

## 3 Conclusions

In summary, a mesoporous Ti_3_C_2_T*_x_*/Ni/AS composite with porosity around 50% was synthesized for simultaneously achieving high MA and mechanical properties. The EMT zeolite was selected as precursor to realize low temperature densification for protecting the Ti_3_C_2_T*_x_* MXene from thermal damage. After densification, the Ti_3_C_2_T*_x_* nanosheets show strong anisotropy alone the in‐plane direction due to the transient viscous flow of the EMT derived glass powder. The alignment of MXene combined with mesoporous glass structure favors the impedance matching because of the low electrical conductivity in the out‐of‐plane direction. Moreover, taking advantage of the ion exchanging capacity of EMT, isolated and uniformly distributed Ni NPs can be incorporated to the AS glass matrix as well, which plays an important role in attaining high attenuation capability while maintaining the good impedance matching. As a result, The Ti_3_C_2_T*_x_*/Ni/AS composite exhibited excellent MA properties with RL_min_ of −59.5 dB at 10.8 GHz and EAB of 4.1 GHz in Ku band. Meanwhile, the composite also shows excellent mechanical properties in terms of compressive strength, flexural strength, hardness and contact‐damage‐resistance, which enable the mesoporous composite to be applied as a structural MAC of high‐performance.

## 4 Experimental Section

The detailed synthesis procedure and characterization can be found in supporting information.

## Conflict of Interest

The authors declare no conflict of interest.

## Supporting information

Supporting Information

## Data Availability

Research data are not shared.
