# Supplementary material for: A Robust Hierarchical MXene/Ni/Aluminosilicate Glass Composite for High‐Performance Microwave Absorption
Source: Adv Sci (Weinh). 2021 Dec 13;9(4):2104163. doi: 10.1002/advs.202104163 (PMC8811826; doi:10.1002/advs.202104163)
Supplement: Supplementary file 1 — Supporting Information [file ADVS-9-2104163-s001.pdf]

WILEY-VCH

ADVANCED SCIENCE
Open Access

Supporting Information

for *Adv. Sci.*, DOI: 10.1002/advs.202104163

A Robust Hierarchical MXene/Ni/aluminosilicate Glass Composite for High-Performance Microwave Absorption

Wei Luo, Mengya Wang, Kangjing Wang, Peng Yan, Jilong Huang, Jie Gao, Tao Zhao, Qi Ding, Pengpeng Qiu, Haifeng Wang, Ping Lu, Yuchi Fan*, Wan Jiang**

Supporting Information

A Robust Hierarchical MXene/Ni/aluminosilicate Glass Composite for High-Performance Microwave Absorption

Wei Luo, Mengya Wang, Kangjing Wang, Peng Yan, Jilong Huang, Jie Gao, Tao Zhao, Qi Ding, Pengpeng Qiu, Haifeng Wang, Ping Lu, Yuchi Fan*, Wan Jiang**

Prof. W. Luo, Prof. Y. Fan, M. Wang, K. Wang, P. Yan, J. Huang, J. Gao, T. Zhao, Q. Ding, P. Qiu, H. Wang, Prof. W. Jiang
State Key Laboratory for Modification of Chemical Fibers and Polymer Materials, College of Materials Science and Engineering, Donghua University, Shanghai 201620, China
E-mail: wluo@dhu.edu.cn (W. Luo); yuchifan@dhu.edu.cn (Y. Fan); wanjiang@dhu.edu.cn (W. Jiang)
Prof. W. Luo, Prof. Y. Fan, Q. Ding, Prof. W. Jiang
Institute of Functional Materials, Donghua University, Shanghai 201620, China
P. Lu
State Key Laboratory of High Performance Ceramics and Superfine Microstructures, Shanghai Institute of Ceramics, Chinese Academy of Sciences, Shanghai 200050, China

1. SUPPLEMENTARY METHODS

1.1 Chemicals

High-purity Ti_3AlC_2 powder was purchased from Laizhou Kai Kai Ceramic Materials Co., Ltd., China. LiF, HCl, $NiCl_2{\cdot}6H_2O$, $NaAlO_2$, and NaOH were purchased from Shanghai Sinopharm Chemical Reagent Co., Ltd., China. Deionized water was used in all experiments. LUDOX®AS-30 colloidal silica 30 wt% suspension in H_2O were purchased from Sigma-Aldrich, Inc.

1.2 Preparation of $Ti_3C_2T_x$ nanosheets

In a typical process, LiF (0.8 g) and HCl (9 M 20mL) were mixed by magnetic stirring for 5min, then Ti_3AlC_2 powder (1 g) was slowly added into the mixture by stirring for 36 h at 35 ℃. The obtained mixture was washed by repeated centrifugation with deionized water until pH reached 6, finally the as prepared dark-green supernatant and colloidal solution of delaminated $Ti_3C_2T_x$ flakes were carefully collected.

1.3 Synthesis of EMT and hybrid powders

EMT was synthesized by a template-free Na_2O–Al_2O_3–SiO_2–H_2O precursor system[1]. The ion-exchange was performed in EMT in order to remove Na^+, and the detailed steps were as follows: 3g EMT was dissolved into NH_4Cl solution (0.5M 75ml) with stirring for 1h, EMT powder was obtained followed by centrifugation and freeze-dried.

The procedures for preparation of $Ti_3C_2T_x$@EMT was almost the same as that for preparation of EMT described above, except that adding certain amount of concentrated $Ti_3C_2T_x$ dispersion into EMT precursor suspension kept in the Ar atmosphere at 30℃.

For EMT-Ni was synthesized by ion-exchange method. In this process, adding EMT powder into $NiCl_2$ solution (0.02M) with stirring to achieve the EMT loaded Ni^{2+}. Then heated at 550 ℃ in Ar/H_2 in order to reduce Ni^{2+} to Ni^0 nanoparticles. $Ti_3C_2T_x$@EMT-Ni powder was prepared via the similar method, except that the amount of $NiCl_2$ was increased from 20, 30 to 40 wt% to achieve different loads of $NiCl_2$ in the $Ti_3C_2T_x$@EMT.

1.4 Sintering of composites

The powder was consolidated by SPS at 700 °C in a vacuum (5 Pa) with the heating rate of 100 °C min^{-1} under uniaxial pressure of 50 MPa, then naturally cooling down to room temperature. The AS glass, Ni/AS and $Ti_3C_2T_x$/AS composites were sintered using the same condition. The ring-like samples with inner and outer diameters of 3.04 and 7.0 mm respectively for electromagnetic properties testing were prepared by the same procedure of sintering using a specially designed die including a pair of hollowed punches with an inserted

stick, while for mechanical testing the samples were sintered by using a die with diameter of 10 mm.

1.5 Characterization

Powder XRD data was collected by Rigaku D/MAXRC X-ray diffractometer with a Cu Kα radiation source (40 kV, 200 mA). The apparent density was calculated by using the weight and geometric volume. SEM images were obtained on a TESCAN VEGA3 equipment. TEM observation and EDS mapping were performed on FEI Talos F200S equipment operating at an accelerating voltage of 200kV. The N_2 adsorption-desorption isotherms were measured by Quantachrome Autosorb-IQ instrument. The specific surface area was calculated based on the Brunauer-Emmett-Teller (BET) method using the adsorption data in a relative pressure (P/P_0) ranged from 0.005 to 0.25. The pore size distribution and pore volume were calculated from the adsorption branches of isotherms using the Barrett-Joyner-Halenda (BJH) method. XPS measurements were performed using a Thermo Scientific ESCALAB 250Xi electron spectrometer (Thermo Electron, America) with an Al Kα1486.6 eV excitation source. The electromagnetic properties of samples were measured at room temperature by the coaxial method in the frequency range of 2−18 GHz using a network analyzer (Keysight PAN-L N523B network analyzer). The reflection loss was obtained by the following equation using the Z_{in}:

$$\mathrm{RL} = 20\left|log\frac{Z_{in}-1}{Z_{in}+1}\right| \qquad (1)$$

Advanced ultrasonic material characterization system (UMS-100, France) was used to measure the Young’s modulus and Poison's ratio. Vickers hardness was determined by the indentation method using a load of 5 Kg for 10 s. Indentation fracture toughness was calculated by the Anstis equation as follows

$$K_{IC} = 0.016(E/H_v)^{0.5}PC^{-1.5} \qquad (2)$$

where E is Young’s modulus, H_V is Vickers indentation hardness, P is the load, and C is half crack length. Modified small punch (MSP) method was used to calculate the strength of monolithic alumina MWCNTs/alumina composite by the following equation:

$$\delta = {}^{3P}\!/_{2\pi t^2}\ [1-[(1-\gamma^2)/4)].\left({}^{b^2}\!/_{a^2}\right)+(1+\gamma)\ln({}^{a}\!/_{b})] \qquad (3)$$

where P is the load, the thickness of the sample is t, γ is the Poison’s ratio, a and b are the radius of the hole of the mold and the radius of the tip of cylindrical punch, respectively.

2. SUPPLEMENTARY FIGURES

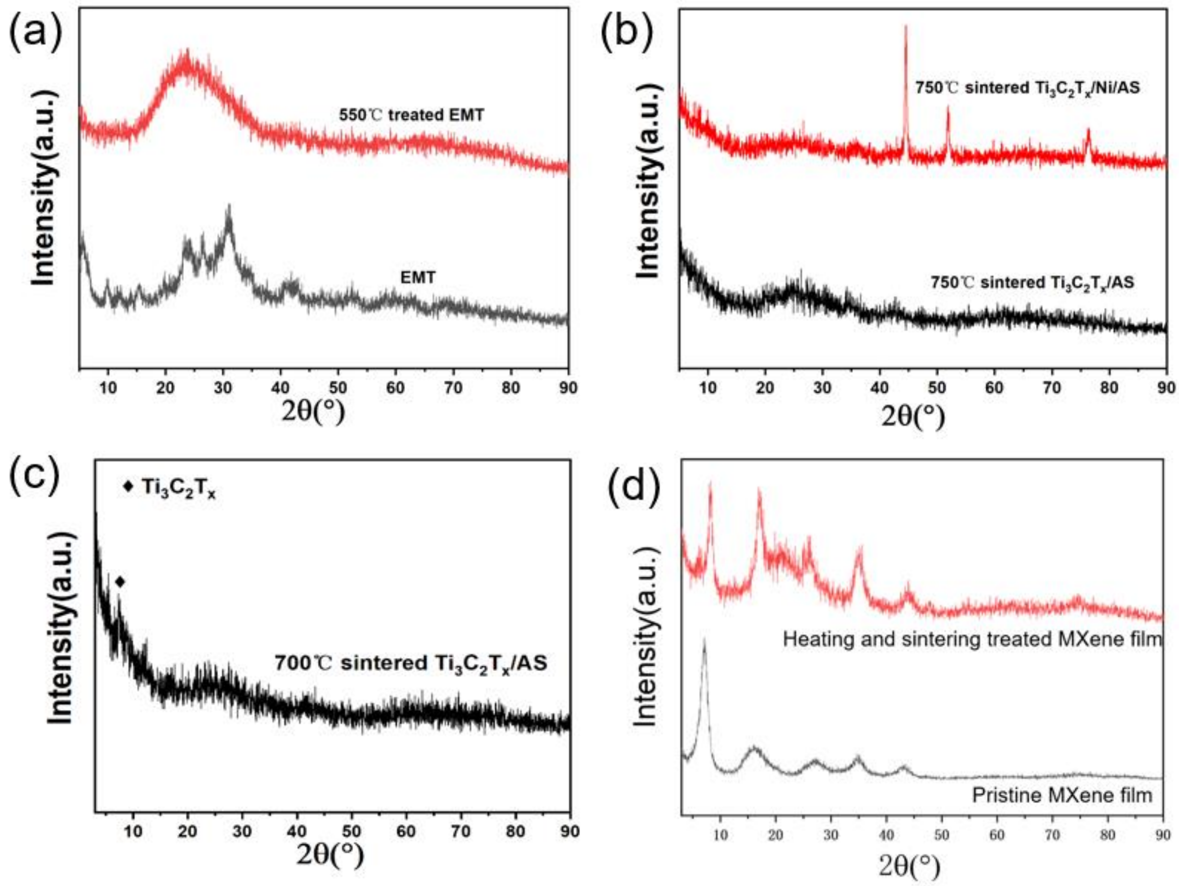

Figure S1. XRD patterns of (a) EMT and heat treated EMT powder at 550°C, (b) sintered $Ti_3C_2T_x$/AS and $Ti_3C_2T_x$/Ni/AS composites sintered at 750°C, (c) $Ti_3C_2T_x$/AS composite sintered at 700°C, and (d) pristine $Ti_3C_2T_x$ film and heating and sintering treated one using the identical conditions for composite processing. The films in (d) were fabricated by vacuum filtration using the same MXene nanosheets in this study.

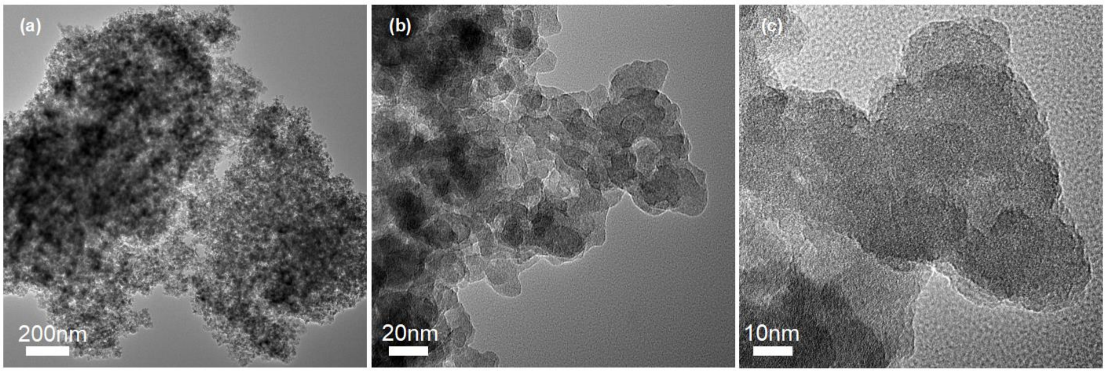

Figure S2. TEM images of pure AS powder treated at 550°C.

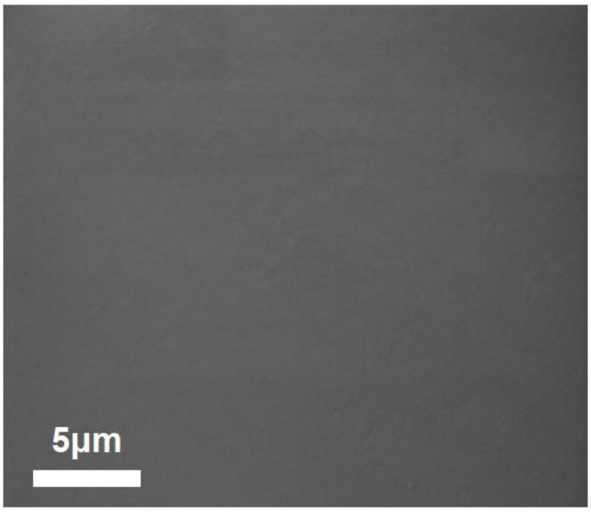

Figure S3. SEM images of fractured surface for fully dense AS glass sintered at 780℃.

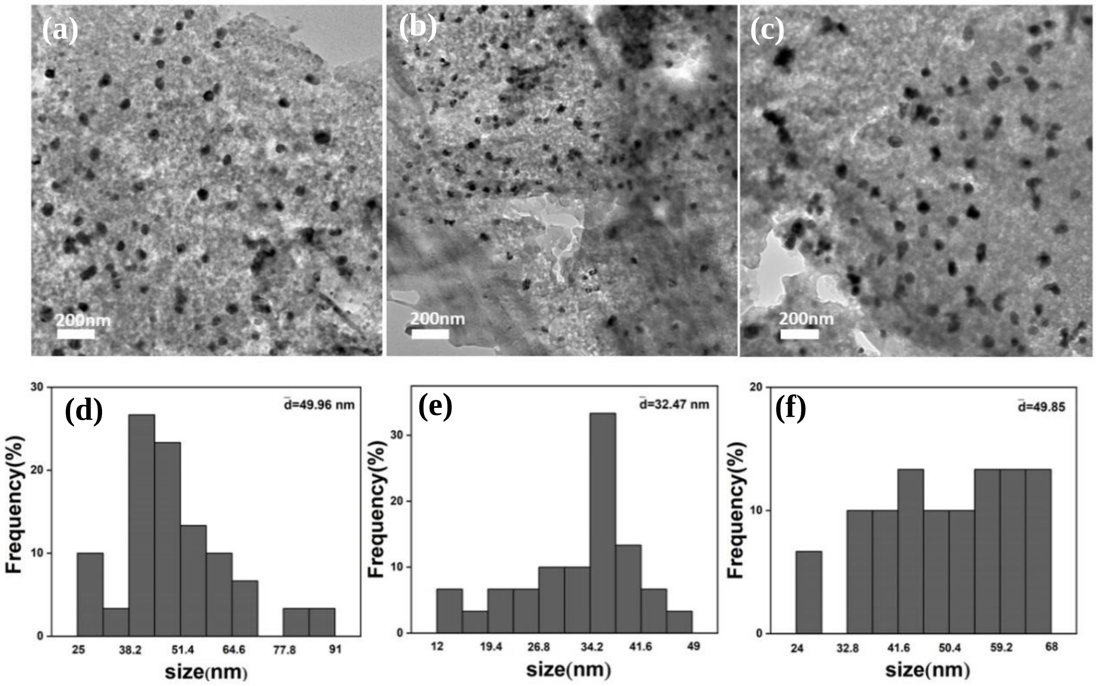

Figure S4. (a-c) TEM images showing the Ni nanoparticles in $Ti_3C_2T_x$/Ni/AS composites with Ni content of 1.8, 3.1, and 4.7 vol%, respectively. (c-f) Corresponding grain size distribution in (a-c), respectively.

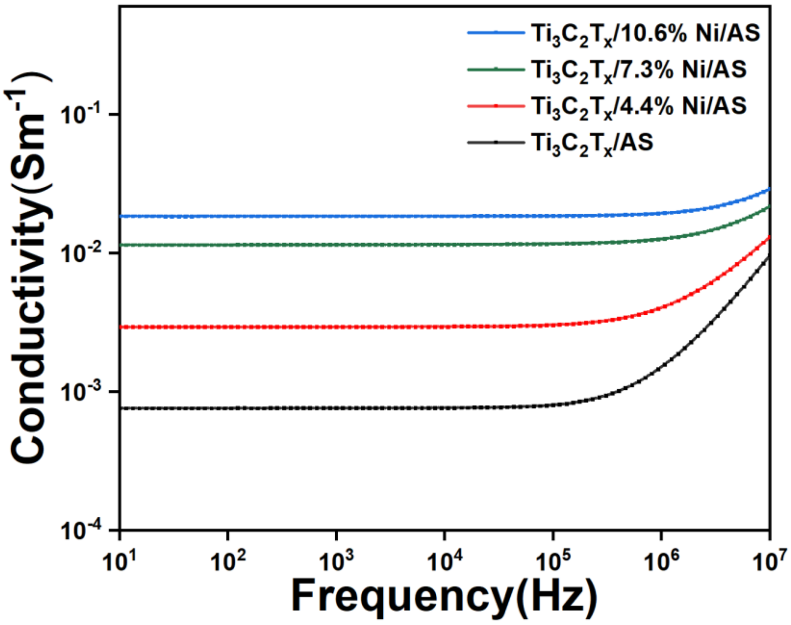

Figure S5. AC electrical conductivity at low frequency range for Ti_3AlC_2/AS and Ti_3AlC_2/Ni/AS composites with various Ni content.

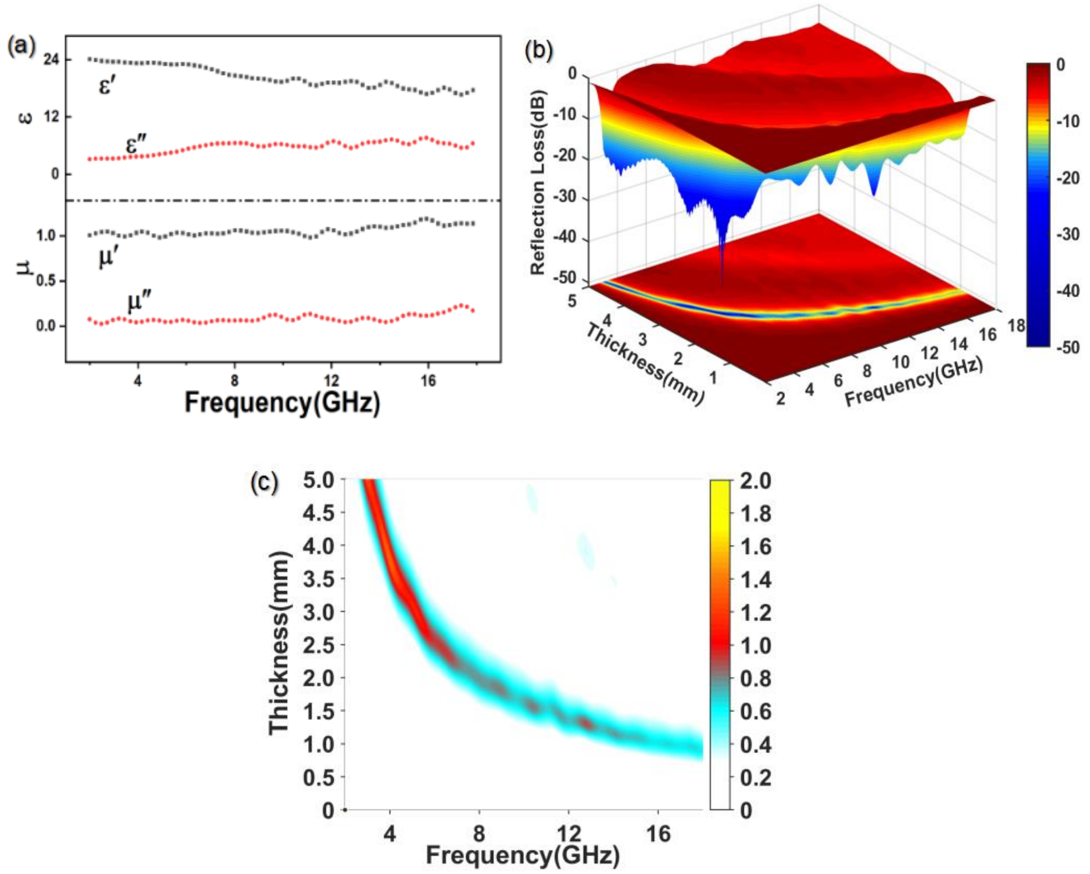

Figure S6. (a) Complex permittivity and permeability, (b) three-dimensional maps of the RL values, and (c) contour map of the values of Z_{in} for 15 wt% $Ti_3C_2T_x$/AS composite.

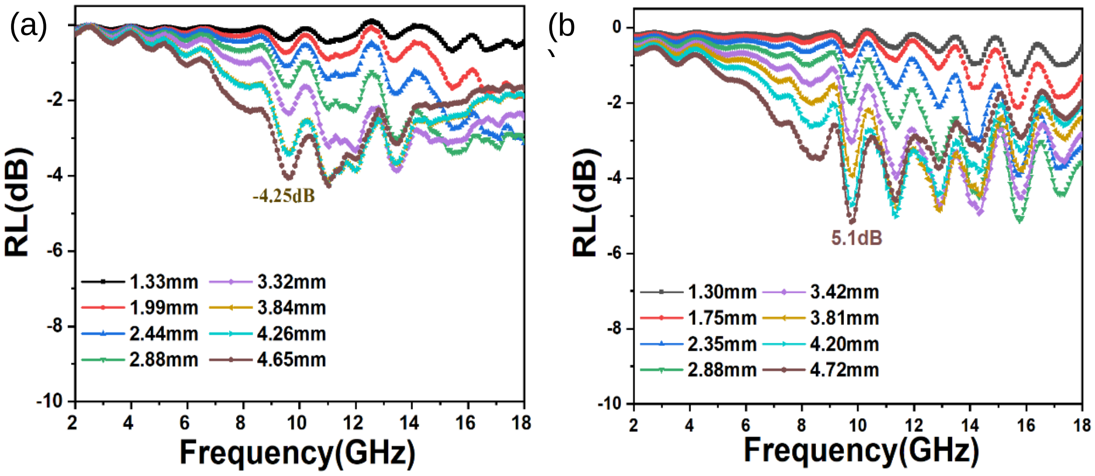

Figure S7. The RL curves of (a) AS glass and (b) Ni/AS composite under different fitting thicknesses.

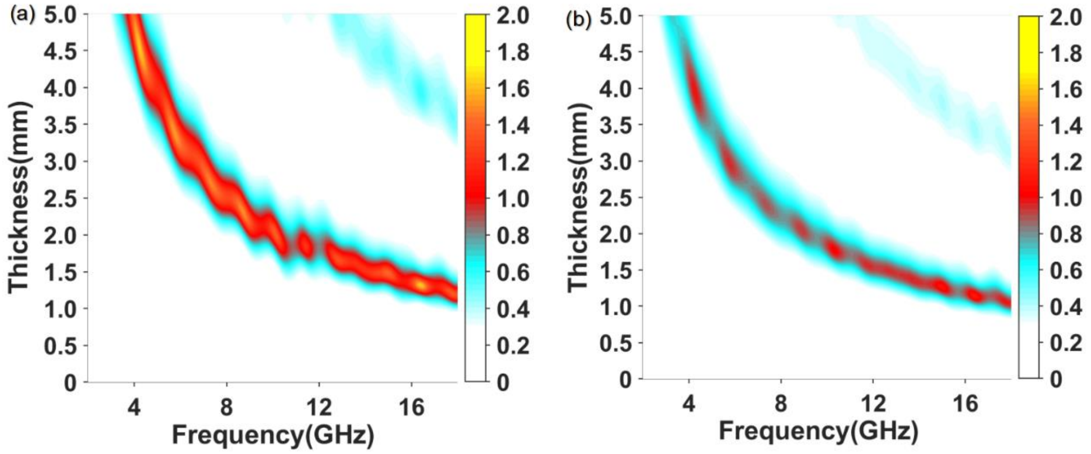

Figure S8. Contour maps of Z_{in} of Ti_3AlC_2/Ni/AS composites with Ni content of 1.8 and 4.7 vol.

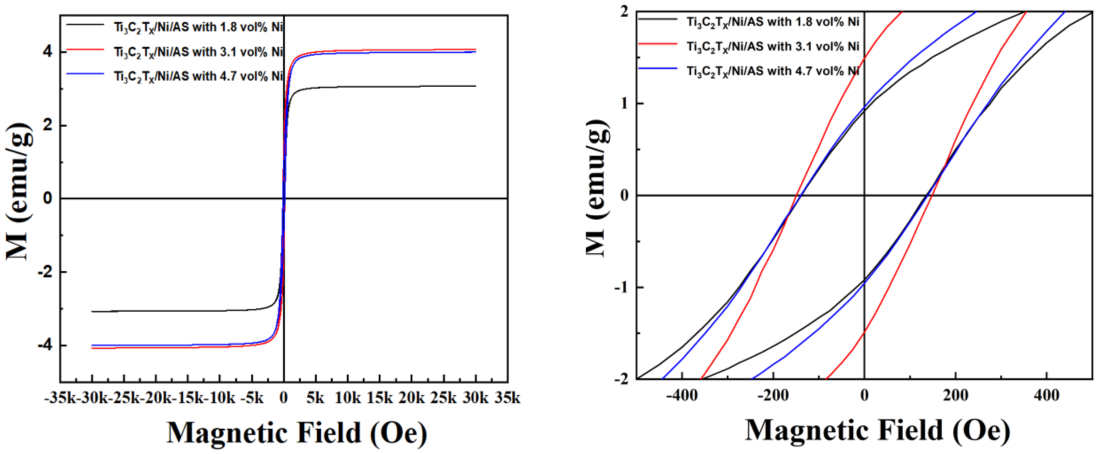

Figure S9. Magnetic hysteresis loops for various composites.

3. SUPPLEMENTAL TABLES

Table S1. Basic information for various samples

Sample	Theoretical Density (gcm^{-3})	Apparent Density (gcm^{-3})	Relative density	In-plane electrical conductivity (Sm^{-1})	Poisson's Ratio	Young's Modulus (GPa)
AS	2.52	1.26	50%	-	0.32	10.2
Ni/AS	2.733	1.46	53.4%	-	0.27	10.33
$Ti_3C_2T_x$/AS	2.57	1.27	49.8%	0.58	0.28	10.72
$Ti_3C_2T_x$ /Ni/AS	2.768	1.51	54.5%	39.09	0.32	10.90

Table S2. Typical MA performance of reported inorganic matrix composites in X band at room temperature.

Filler	Filler content	Matrix	Thickness (mm)	RL_{min} (dB)	EAB	Reference
GN	2.5 vol%	MgO	1.5	-36.5	2	[2]
GN	1.0 vol%	Al_2O_3	1.5	-24	1.6	[3]
CNT	1.56 wt%	$Sc_2Si_2O_7$	2.85	-33.5	4.2	[4]
Graphene	<0.01wt%	Si_3N_4	3.75	-26.7	4.2	[5]
GO	7 wt%	SiO_2	2.33	-25	4.2	[6]
rGO@Fe_3O_4	0.3 wt%	SiBCN	2.15	-43.78	2.06	[7]
SiC_{NW}	-	SiOC	3.3	-20.01	1.6	[8]
SiC	10 wt%	Double-layer LAS	2+2	-32	4.2	[9]
$Ti_3C_2T_x$	8.8 vol%.	AS glass	2.35	-41.65	2.6	This Work
$Ti_3C_2T_x$/Ni	10.9 vol%.	AS glass	2.04	-59.5	3.0	This Work

REFERENCES

[1] N. Eng-Poh, D. Chateigner, T. Bein, V. Valtchev, S. Mintova, *Science.* **2012**, *335*, 70.

[2] C. Chen, L. Pan, S. Jiang, S. Yin, X. Li, J. Zhang, Y. Feng, J. Yang, *J. Eur. Ceram. Soc.* **2018**, *38*, 1639.

[3] Y. Qing, Q. Wen, F. Luo, W. Zhou, *J. Mater. Chem. C.* **2016**, *4*, 4853.

[4] H. Wei, X. Yin, X. Li, M. Li, X. Dang, L. Zhang, L. Cheng, *Carbon.* **2019**, *147*, 276.

[5] F. Ye, Q. Song, Z. Zhang, W. Li, S. Zhang, X. Yin, Y. Zhou, H. Tao, Y. Liu, L. Cheng, L. Zhang, H. Li, *Adv. Funct. Mater.* **2018**, *28*, 1707205.

[6] M. S. Cao, X. X. Wang, W. Q. Cao, X. Y. Fang, B. Wen, J. Yuan, *Small.* **2018**, *14*, 1800987.

[7] C. J. Luo, T. Jiao, J. W. Gu, Y. S. Tang, J. Kong, *Acs Appl Mater Inter.* **2018**, *10*, 39307.

[8] W. Y. Duan, X. W. Yin, Q. Li, X. M. Liu, L. F. Cheng, L. T. Zhang, *J. Eur. Ceram. Soc.*

2014, *34*, 257.

[9] C. H. Peng, P. S. Chen, C. C. Chang, *Ceram. Int.* **2014**, *40*, 47.
